# SIRT1 Catalytic Activity Has Little Effect on Tumor Formation and Metastases in a Mouse Model of Breast Cancer

**DOI:** 10.1371/journal.pone.0082106

**Published:** 2013-11-21

**Authors:** Katherine V. Clark-Knowles, Danielle Dewar-Darch, Karen E. Jardine, Michael W. McBurney

**Affiliations:** 1 Centre for Cancer Therapeutics, Ottawa Hospital Research Institute, Ottawa, Ontario, Canada; 2 Department of Medicine, University of Ottawa, Ottawa, Ontario, Canada; Baylor College of Medicine, United States of America

## Abstract

The protein deacetylase SIRT1 has been implicated in the regulation of a large number of cellular processes that are thought to be required for cancer initiation and progression. There are conflicting data that make it unclear whether *Sirt1* functions as an oncogene or tumor suppressor. To assess the effect of SIRT1 on the emergence and progression of mammary tumors, we crossed mice that harbor a point mutation that abolishes SIRT1 catalytic activity with mice carrying the polyoma middle T transgene driven by the murine mammary tumor virus promoter (MMTV-PyMT). The absence of SIRT1 catalytic activity neither accelerated nor blocked the formation of tumors and metastases in this model. There was a lag in tumor latency that modestly extended survival in *Sirt1* mutant mice that we attribute to a delay in mammary gland development and not to a direct effect of SIRT1 on carcinogenesis. These results are consistent with previous evidence suggesting that *Sirt1* is not a tumor promoter or a tumor suppressor.

## Introduction

 The lifespan of many metazoan animals can be prolonged by restricting daily caloric intake [1]. The working model emerging from these studies is that caloric restriction (CR) activates the catalytic activity of a class of enzymes called sirtuins and this is responsible for conferring stress resistance and extending lifespan [2–4]. Sirtuins are NAD^+^-dependent protein deacetylases [5] and the most studied mammalian member of this group is SIRT1. 

 In mammals, CR extends lifespan and forestalls the onset of various diseases including cancer [1]. There is evidence that *Sirt1* is required for CR-induced lifespan extension in mice [6] suggesting that *Sirt1* may be a tumor suppressor gene. Indeed a number of studies have suggested that this is true [7–10] although paradoxically there are as many studies indicating that *Sirt1* is an oncogene [11–15] and still others demonstrating that *Sirt1* has no effect on oncogenesis [16]. Most reviews on the subject describe *Sirt1* as having both pro- and anti-oncogenic properties [17]. 

*Sirt1* is a promiscuous protein deacetylase with more than 80 established substrates [18] and a large number of other proteins with which it interacts [19]. Amongst its substrates are a number of well-established proteins with roles in the initiation and progression of cancer. These include p53 [5,12], p73 [20], RB [21], NF-κB [22], and c-MYC [23]. SIRT1 itself is in turn regulated by tumor suppressor proteins including HIC1 [13], BRCA1 [24], and the putative tumor suppressor DBC1 [25,26]. The notion of a major role for SIRT1 in carcinogenesis is further strengthened by the apparent involvement of SIRT1 in the maintenance of genome stability [9,27]. 

 A potential role for *Sirt1* in the etiology of breast cancer was postulated recently when a retrospective study found that breast cancer patients whose tumors where positive for SIRT1 via immunohistochemistry had an increased likelihood of metastasis to a distant site as well as decreased overall survival and relapse-free survival [28,29]. SIRT1 expression has also been found to correlate with metastatic spread in the triple negative subtype [29]. A putative SIRT1 activator was found to promote the formation of lung metastases in a breast cancer xenograft model [30]. SIRT1 expression is decreased in breast cancer arising in BRCA1 mutation carriers [24].

 Our previous studies of cancer development employed animals carrying a null mutation for *Sirt1* and used 2-stage skin carcinogenesis and APC-dependent colon cancer [16]. These studies showed that the *Sirt1* genotype did not influence the efficiency of cancer development but in both cases, the tumors arising with these 2 paradigms are benign polyps or adenomas that did not progress into frank malignancies. We set out to investigate the possibility that *Sirt1* is involved in later stages of carcinogenesis, using a transgenic mouse (MMTV-PyMT) in which the polyoma middle t antigen is expressed in the mammary epithelium. These animals develop rapidly growing mammary tumors that frequently metastasize to the lung [31]. The MMTV-PyMT mouse model of breast cancer is a long-established and well characterized model that accurately recapitulates the disease process observed in human breast cancer [32]. We introduced the MMTV-PyMT transgene into animals carrying a point mutation in the *Sirt1* gene, the *Sirt1* ^tm2.1Mcby^ gene referred here to as the *Sirt1* ^Y^ allele. This gene encodes a SIRT1(H355Y) protein that has no catalytic activity [33]. This point mutation was created by gene knock-in, and thus the mutant protein is expressed throughout the mice and is present at levels indistinguishable from the SIRT1 levels from the Sirt1^+^ allele. Our results indicate that mice carrying the MMTV-PyMT transgene and homozygous for *Sirt1* ^Y^ efficiently develop mammary tumors that subsequently metastasize.

## Materials and Methods

### Animals

Male FVB/N-Tg(MMTV-PyMT)634Mul/J mice (hereto referred to as MMTV-PyMT), were a generous gift from Dr. Bill Muller [31]. These male animals were crossed with heterozygotes (*Sirt1* ^Y/+^) of our own female 129sv/CD1-Sirt1^tm2Mcby^ mice who harbour a missense mutation in the catalytic domain of *Sirt1* [33]. Male mice from the resulting F1 generation who were positive for the MMTV-PyMT transgene and *Sirt1* ^Y/+^ were then crossed with female *Sirt1* ^Y/+^ mice. From the resulting F2 generation, only female mice that were positive for the MMTV-PyMT transgene were followed. Genotyping for the MMTV-PyMT transgene and the *Sirt1* H355Y mutation was performed as previously described [33,34]. Mice were housed in groups of 2-4, with a constant room temperature of 24°C and a 12 hour light/dark cycle. They received food and water ad libitum. Upon weaning, animals were weighed weekly and digital palpation of the mammary glands was used to assess the presence of palpable masses. Mice were monitored until they had reached criteria for predetermined loss of wellness endpoint. These endpoints were defined as tumor burden where any tumor had a diameter of 20 mm, impaired mobility, tumor ulceration, and/or respiratory distress. All animal work was carried out in accordance with *Guidelines for the Care and Use of Animals* established by the Canadian Council on Animal Care with protocols approved by the Animal Care Committee of the University of Ottawa, Ottawa, Ontario, Canada.

### Tissue Collection

Animals were euthanized via CO_2_ asphyxiation. Tumors were removed, weighed and fixed in 10% neutral buffered formalin. Formalin-fixed tissues were embedded in paraffin and 3-4 µm sections were cut for staining with hematoxylin and eosin or for immunohistochemistry. Lungs were perfused with phosphate buffered saline and fixed in formalin. 

### Lung Metastases

To assess the presence and degree of metastasis to the lungs, lung tissue was collected as described above. Following embedding in paraffin, eight 10 µm sections, spaced at 50 µm intervals, where cut and affixed to glass slides. Sections were deparaffinized and stained with hematoxylin and eosin. Slides were blinded and the total number of individual metastatic nodules in each sample of lungs was counted at 100X magnification using an Olympus BX50 microscope (Olympus, Melville, NY, USA).

### Mammary Gland Whole Mounts

At six or eleven weeks of age, virgin female mice were euthanized via carbon dioxide asphyxiation and the fourth abdominal mammary gland was dissected, spread onto a glass slide and allowed to dry for 30 minutes. Slides were placed in acetone overnight followed with hematoxylin for 4 hours. Glands were then destained in an acid-alcohol solution overnight and then dehydrated in 100% ethanol for 1 hour followed by transfer to xylene. Whole mounts were then coverslipped with Permount. Mammary glands from ten mice from each genotype were examined at each time point.

### Immunohistochemistry

Paraffin sections were deparaffinised through three changes of xylene and rehydrated in series of graded ethanols. High temperature antigen retrieval was performed using a 0.01M sodium citrate buffer (pH 6.0) in PBS and endogenous peroxidase activity was blocked via treatment with 3% hydrogen peroxide in PBS. Additional blocking was performed using a serum-free protein block (DAKO, Carpenteria, CA, USA). Primary antibodies were diluted in background-reducing antibody diluent (DAKO) at the following concentrations: SIRT1, 1:50 (Cell Signaling Technologies, Danvers, MA, USA), Middle T Antigen, 1:15 (Ab-4, Calbiochem, Mississauga, ON, Canada), ERα, 1:100 (MC-20, Santa Cruz Biotechnology, Santa Cruz, CA, USA ). For the SIRT1 and ERα antibodies, sections were incubated with primary antibody overnight at room temperature. Following three washes with PBS, sections were incubated with an anti-rabbit Envision+ Labelled Polymer (Dako) for 30 minutes at room temperature. For the polyoma Middle T Antigen antibody the Vector®Mouse on Mouse™ kit (Vector Labs, Burlingame, CA, USA) was followed according to the manufacturer’s specifications. Developing was performed with diaminobenzidine (DAB, Sigma-Aldrich, Oakville, ON, Canada) and slides were counterstained with hematoxylin, dehydrated and coverslipped using Permount (Fisher Scientific, Ottawa, ON, Canada)

### Statistics

The probability of significant differences was determined by analysis of variance (ANOVA), employing the Kruskal-Wallis test with the Dunn’s multiple comparison test. Survival and time-to-detection curves were compared using the LogRank test. Correlation was tested using the Spearman rank test. Data is expressed as mean±SEM (standard error of the mean) and *P*-values are two-sided. Analysis was performed using Graphpad Prism statistical software (Graphpad Software, San Diego, CA, USA).

## Results

### Abrogation of SIRT1 catalytic activity does not prevent mammary tumor formation in MMTV-PyMT mice

**Figure 1:**
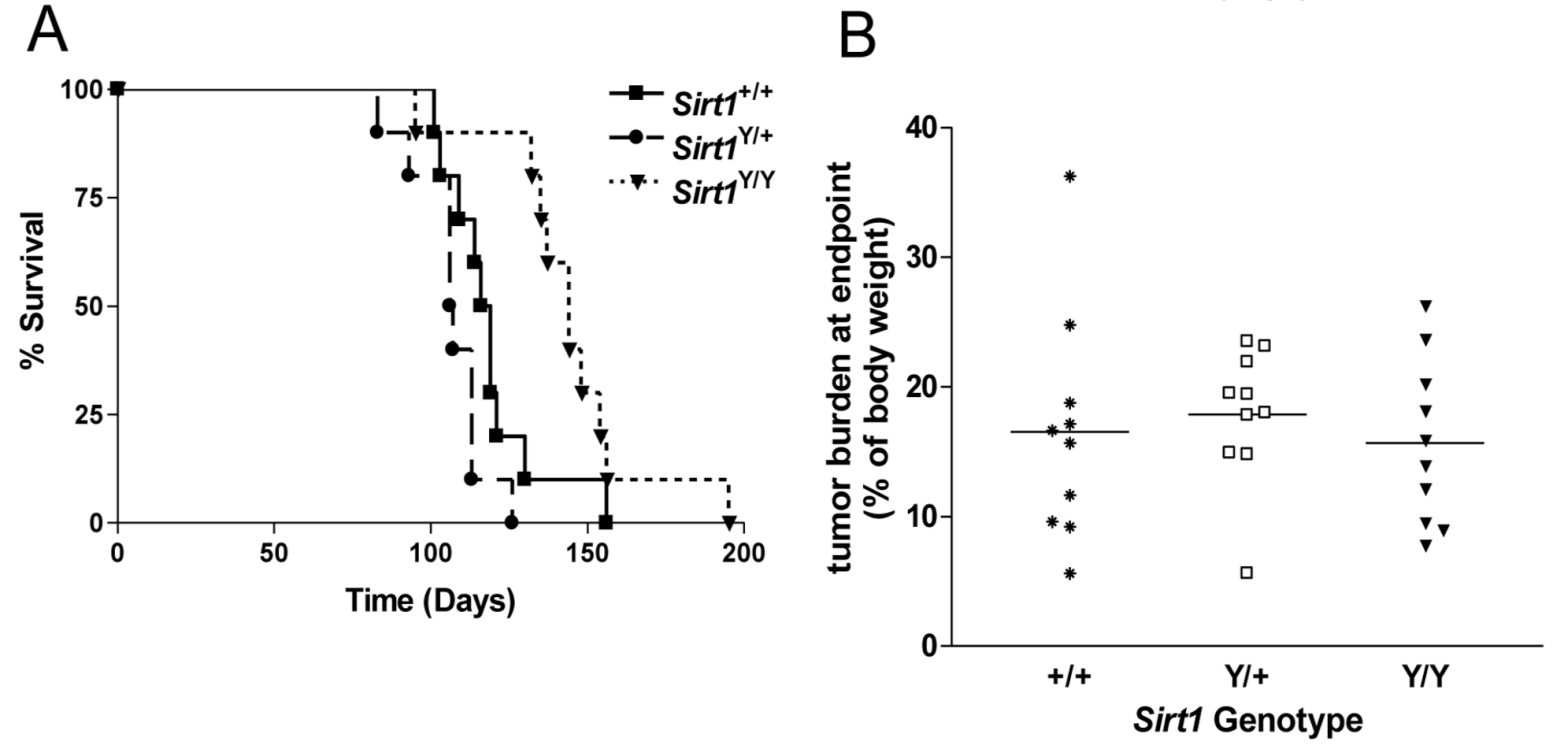
Abrogation of SIRT1 catalytic activity does not prevent mammary tumor formation in the MMTV-PyMT mouse model of breast cancer. **A**) Kaplan Meir plot showing the percentage of surviving animals over time. N= 10 mice per genotype. *Sirt1* ^Y/Y^ animals had a significantly longer overall survival time than the *Sirt1* ^+/+^ and the *Sirt1* ^Y/+^ mice (p <0.01) **B**) Tumor burden as a proportion of total body weight at humane endpoint. All tumors were removed and weighed at necropsy. Points represent individual animals and bars represent the median. N=10 mice per genotype, all animals carried the MMTV-PyMT transgene.

**Figure 2:**
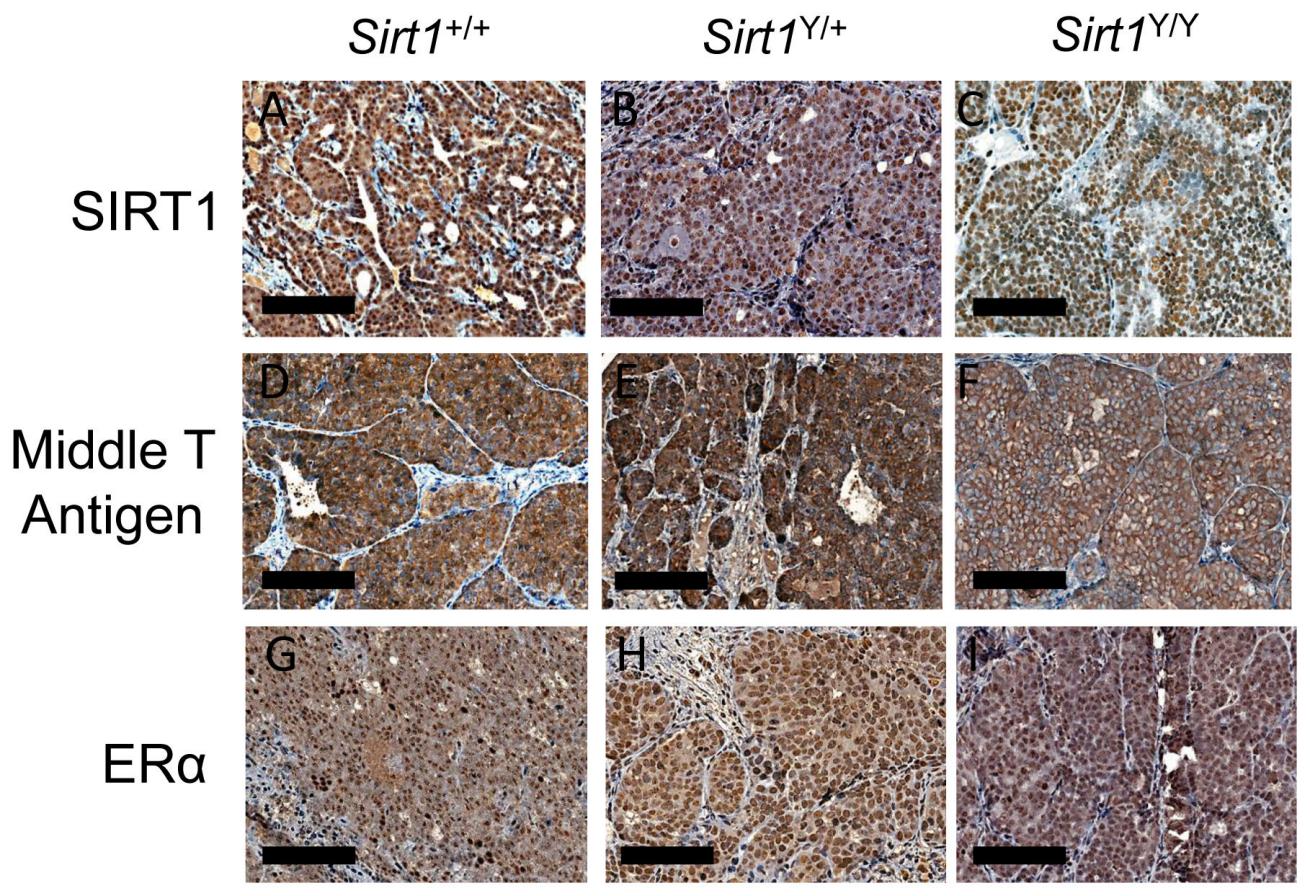
Expression of SIRT1, Middle T Antigen, and ERα protein in mammary tumors. Representative immunohistochemical staining for SIRT1 (A-C), Middle T Antigen (D-F), and ERα (G-I) in mammary tumors collected at humane endpoint from *Sirt1* ^+/+^, *Sirt1* ^Y/+^ and *Sirt1* ^Y/Y^ mice at 200X magnification (scale bars, 100 μm).

 Female mice carrying the MMTV-PyMT transgene develop mammary tumors that progress rapidly and metastasize to the lung [31]. We introduced the MMTV-PyMT transgene into stocks of mice carrying the H355Y point mutation in the *Sirt1* gene (an allele referred to as *Sirt1* ^Y^) and studied the emergence of mammary tumors in *Sirt1* ^*+/+*^, *Sirt1* ^*+/Y*^, and *Sirt1* ^*Y/Y*^ females. All females regardless of *Sirt1* genotype developed tumors that required the sacrifice of the animal within 200 days of birth (Figure 1A). Upon necropsy, all mammary tumors were removed, weighed and expressed as a proportion of body weight. The total tumor burden at endpoint was similar for the 3 genotypes (Figure 1B). Representative mammary tumors were examined histologically. All were positive for expression of the polyoma middle T antigen, which was associated with the cytoplasmic membrane (Figure 2D-F), and for estrogen receptor alpha (ERα), which was present in the nucleus (Figure 2G-I). The SIRT1 protein was also present in the nuclei of cells from all tumors regardless of genotype (Figure 2A-C).

### Loss of SIRT1 catalytic activity is associated with increase tumor latency in MMTV-PyMT mice

**Figure 3:**
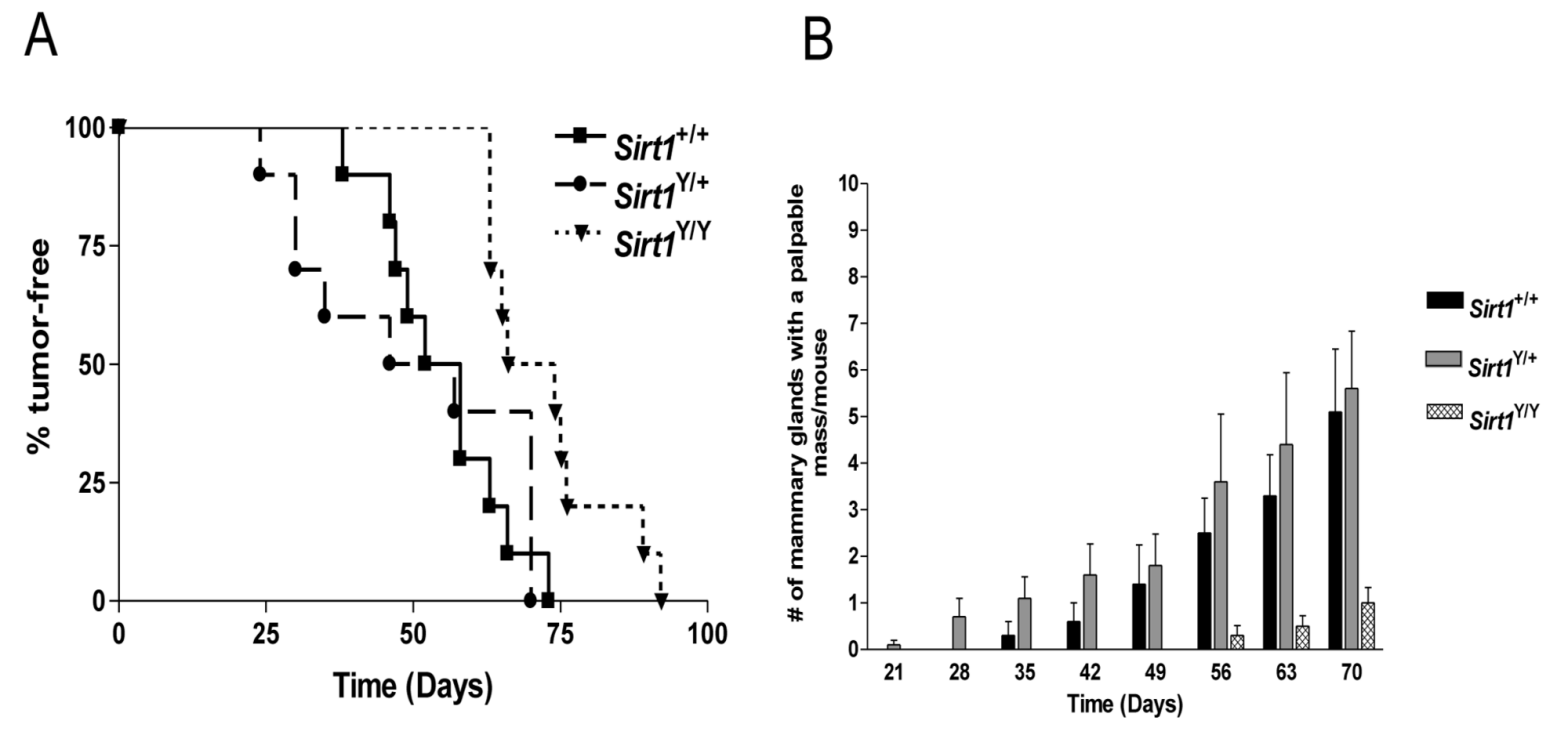
Loss of SIRT1 catalytic activity is associated with increase tumor latency **A)** Kaplan Meir plot measuring the percentage of mice without any palpable mammary gland mass at the given age. N= 10 mice per genotype. There was a significant delay in the time at which the *Sirt1* ^Y/Y^ developed their first detectable mass as compared to the *Sirt1* ^+/+^ and the *Sirt1* ^Y/+^ mice (P <0.01 and P < 0.05, respectively). **B)** The mean number of mammary glands with a palpable mass over time as measured at weekly intervals after birth. N= 10 mice per genotype. Error bars indicate SEM.

 Most of the *Sirt1* ^Y/Y^ mice reached endpoint later than their *Sirt1* ^+/+^ and *Sirt1* ^+/Y^ littermates (Figure 1A). Median survival of *Sirt1* ^Y/Y^ animals was 144 days (range: 95-195 days), which was significantly longer than that of the *Sirt1* ^+/+^ animals (median 117.5 days, range: 101-156 days, P< 0.05) as well that of the *Sirt1* ^Y/+^ mice (median 106 days, range: 83-126 days, P<0.0001). There was no statistically significant difference in the overall survival of the *Sirt1* ^+/+^ and the *Sirt1* ^Y/+^ animals. We monitored the animals at weekly intervals and assessed the age at which the first palpable mammary tumor was detected. Palpable tumors appeared in the *Sirt1* ^Y/Y^ mice with significantly longer latency than those of *Sirt1* ^*+/+*^ and *Sirt1* ^*+/Y*^ mice (Figure 3A). The median age of detection of the first mammary gland mass was 55 days (range: 38-73 days) in *Sirt1* ^+/+^ mice and 57 days (range: 24-70 days) in *Sirt1* ^Y/+^ animals. Both were significantly shorter than the 70 days (range: 63-92 days) observed in *Sirt1* ^Y/Y^ mice (P< 0.01). The delayed onset of tumor development in *Sirt1* ^Y/Y^ animals was more obvious (Figure 3B) when we counted the number of mammary glands that had a palpable mass (there are 10 mammary glands on each mouse). At ten weeks of age, when all ten animals in each group were still alive, the *Sirt1* ^Y/Y^ animals had significantly fewer palpable tumors than either the *Sirt1* ^+/+^ (1.0±1.0 versus 5.1±1.3, P< 0.05) or *Sirt1* ^Y/+^ mice (1.0±1.0 versus 5.6±1.2, P< 0.01). 

**Figure 4:**
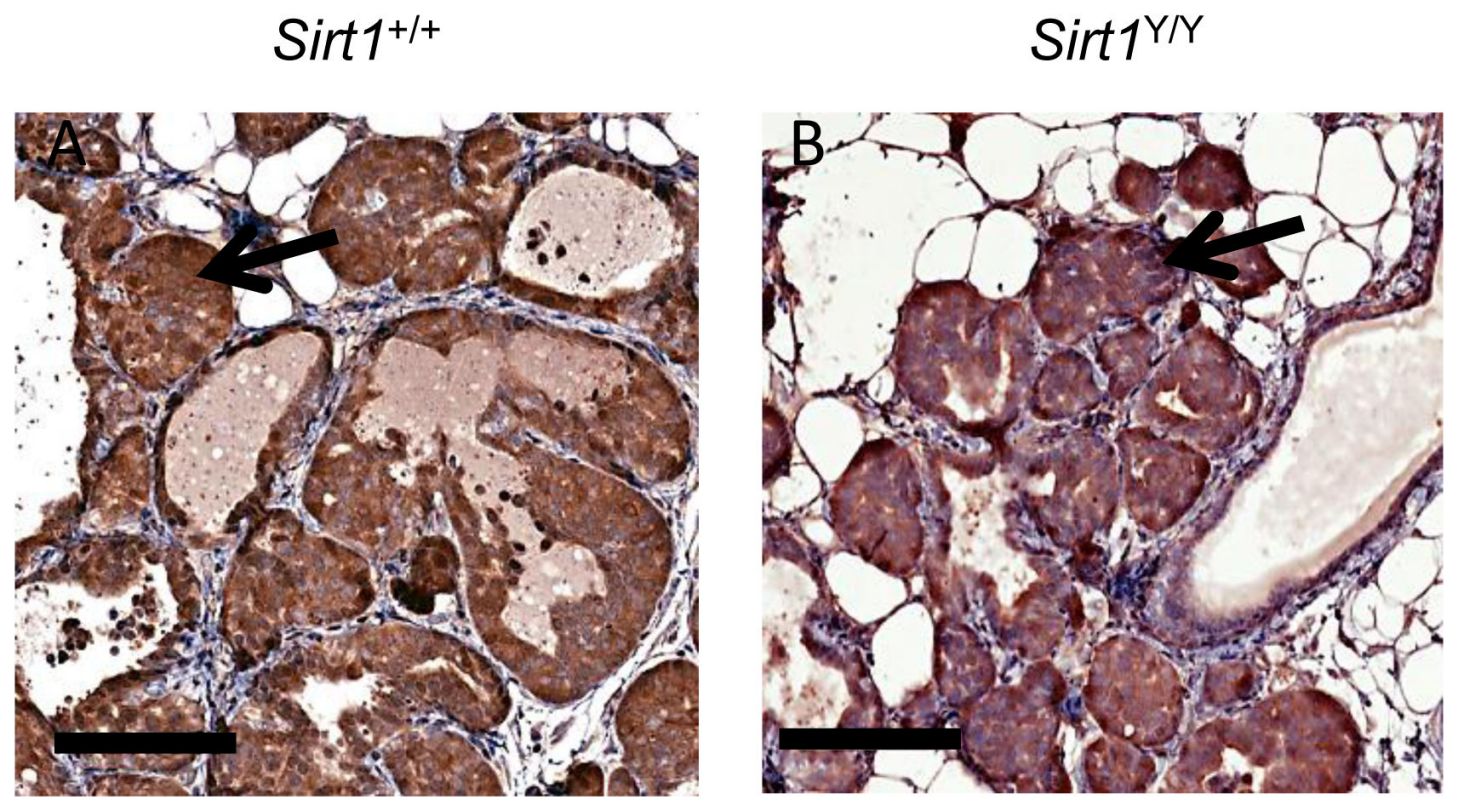
Loss of SIRT1 catalytic activity does not affect expression of the PyMT transgene. Representative immunohistochemical staining for Middle T Antigen in mammary glands of PyMT^+^/*Sirt1* ^+/+^ and PyMT^+^/*Sirt1* ^Y/Y^ mice collected at 6 weeks of age (scale bars equal to 100 μm). Arrows indicate areas of mammary intraepithelial neoplasia.

 Histological examination of mammary glands of 6 week old animals carrying the MMTV-PyMT transgene indicated similar levels of polyoma middle T antigen in both *Sirt1* ^+/+^ and *Sirt1* ^Y/Y^ mice indicating that loss of SIRT1 catalytic activity did not impact the expression of the PyMT oncogene (Figure 4). These sections also revealed microscopic pre-neoplastic hyperplastic nodules in all animals examined regardless of *Sirt1* genotype suggesting that the delay in tumor development in the *Sirt1* ^*Y/Y*^ mice was a consequence of reduced rates of tumor progression rather than initiation. 

### Delayed mammary gland development in *Sirt1* ^Y/Y^ mice

**Figure 5:**
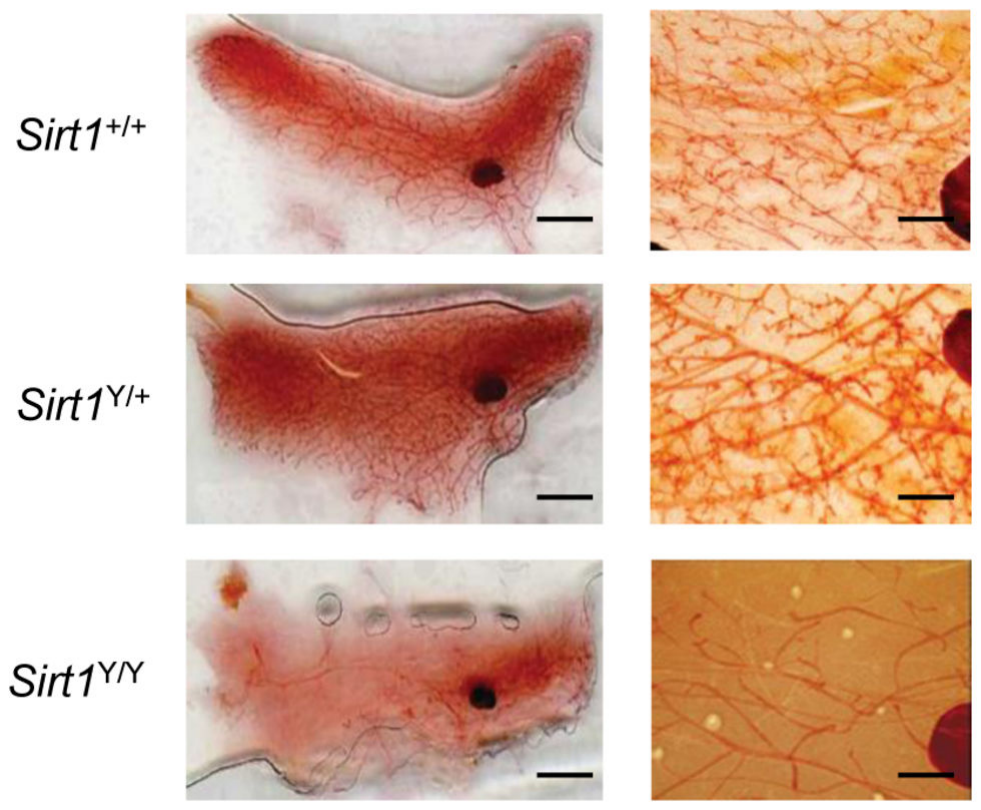
Abolition of SIRT1 enzymatic activity results in blunted ductal morphogenesis in the mammary gland. Right panel, representative photographs of whole mounts of the 4^th^ abdominal mammary gland in *Sirt1* ^+/+^, *Sirt1* ^Y/+^ and *Sirt1* ^Y/Y^ mice at eleven weeks of age (scale bar, 5mm). Left panel, a higher magnification view of the ductal network in representative mammary gland whole mounts of the 4^th^ abdominal mammary gland in *Sirt1* ^+/+^, *Sirt1* ^Y/+^ and *Sirt1* ^Y/Y^ mice at eleven weeks of age. 400X magnification (scale bar, 0.7 mm).

 SIRT1 is reported to be required for efficient maturation of the mammary gland [35]. We examined the fourth inguinal mammary gland from virgin mice at eleven weeks of age, when development should be complete. These mice did not carry the MMTV-PyMT transgene. The *Sirt1* ^+/+^ and *Sirt1* ^Y/+^ animals had mammary glands comprised of a complex ductal network with primary and secondary branching throughout the length of the gland (Figure 5). In all cases examined, the mammary glands from *Sirt1* ^Y/Y^ mice appeared to have stunted branching ductal morphogenesis. There was incomplete outgrowth into the entire gland and the ductal branching structure was simplistic when compared to the glands from *Sirt1* ^+/+^ and *Sirt1* ^Y/+^ mice (Figure 5). 

### SIRT1 catalytic activity does not affect metastasis in MMTV-PyMT mice

**Figure 6:**
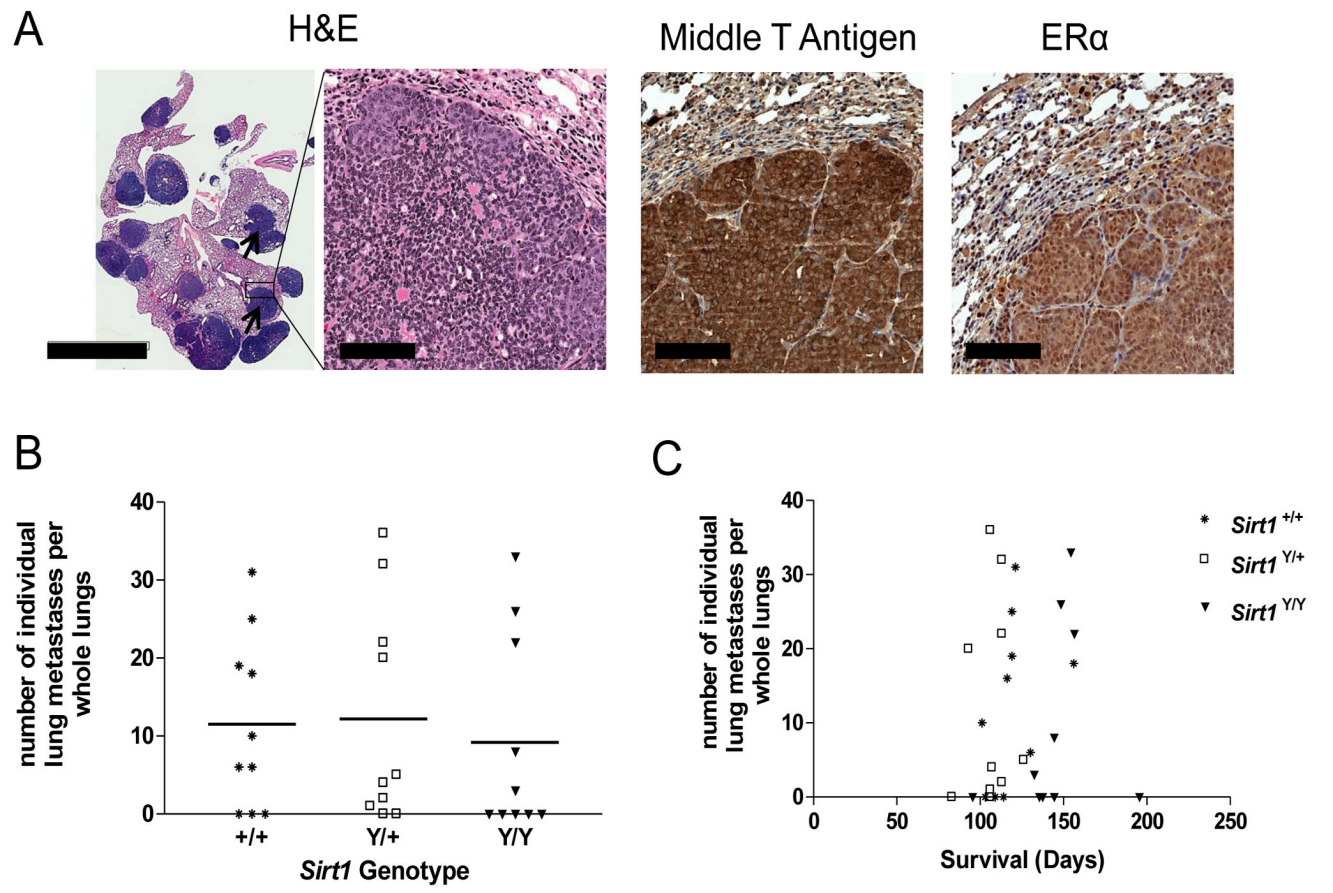
Loss of SIRT1 catalytic activity does not affect metastasis. **A**) Mouse lungs displaying metastatic nodules (arrows) (H&E, left, scale bar 5 mm, right, scale bar 100 μm) and metastatic nodules stained via immunohistochemistry for polyoma middle T antigen and ERα (scale bar 100 μm) **B**) The number of individual metastatic nodules in whole lung H&E sections assessed at endpoint. Eight 10µm sections spaced 50µm apart were evaluated in each mouse. N=10 mice per genotype. Points represent individual animals and bars represent the mean number of metastatic nodules. **C**) The average number of individual metastatic nodules in whole lung H&E sections assessed at endpoint correlated with overall survival time in days.

The mammary tumors that arise in animals carrying the MMTV-PyMT transgene are highly malignant and frequently form lung metastases [31]. In order to determine if SIRT1 affects metastatic spread, lungs were formalin-fixed at endpoint and the presence and number of metastatic nodules per lung were assessed in H&E stained sections (Figure 6A). In our experiments, lung metastases were detected in 70% of the *Sirt* ^*+/+*^, 80% of *Sirt* ^*Y/+*^, and 50% of the *Sirt* ^*Y/Y*^ mice. The number of metastatic nodules was not different (P > 0.05) between animals of the various *Sirt1* genotypes when metastases were present (Figure 6B), with a mean of 11.5±3.5 nodules (range 0-31) observed in the lungs of *Sirt* ^*+/+*^ mice, 12.2±4.2in the *Sirt* ^Y/+^ animals (range 0-36), and 9.2±4.0 in the *Sirt* ^*Y/Y*^ animals (range 0-33). As with the primary mammary tumors, lung nodules expressed both polyoma middle T antigen and ERα (Figure 6A).There was no correlation between the number of lung metastases and survival time observed in the *Sirt* ^*+/+*^ (*r*= 0.58, P=0.09), *Sirt* ^Y/+^ (*r*= 0.36, P=0.30) or the *Sirt* ^*Y/Y*^ mice (*r*= 0.43, P=0.20)(Figure 6C).

## Discussion

 Amongst the multitude of substrates of SIRT1 catalysis [18] and its interacting proteins [36] are many known to play important roles in oncogenesis. The evidence has been generally interpreted to indicate that *Sirt1* is a tumor suppressor gene [37]. We undertook to directly test the idea that the protein deacetylase activity of SIRT1 suppresses tumor formation in mice that develop aggressive mammary carcinomas under the influence of the polyoma middle T oncogene. Tumor development in mice with catalytically inactive SIRT1(H355Y) protein was not accelerated, a result inconsistent with the notion that *Sirt1* is a tumor suppressor. This conclusion is the same as from our previous work in skin and intestinal carcinogenesis [16]. Tumors that arise in MMTV-PyMT mice metastasize to the lung and this late stage of tumor progression is also not accelerated in *Sirt1* ^*Y/Y*^ mice suggesting that SIRT1 has little effect at even late stages of tumor development.

 Mammary glands from 6 week old female *Sirt1* ^*Y/Y*^ mice carrying the MMTV-PyMT transgene contained sites of hyperplasia/adenoma similar to those found in *Sirt1* ^*+/+*^ mice suggesting that SIRT1 has little effect on the very early stages of tumor initiation. However, the rate at which palpable mammary tumors appeared in *Sirt1* ^Y/Y^ animals was delayed compared to that of their normal littermates and the proportion of mice with lung metastases was also slightly reduced. These observations may indicate that SIRT1 has some tumor promoting activity. An alternative explanation for this delay in oncogenesis is that it is a consequence of the delay in the rate of maturation of mammary glands in *Sirt1* ^Y/Y^ animals. Li et al [35] reported impeded ductal morphogenesis in the mammary glands and lactation failure in SIRT1-deficient mammary tissue. We similarly found blunted ductal outgrowth and a less complex ductal network in *Sirt1* ^*Y/Y*^ mice despite the fact that *Sirt1* ^*Y/Y*^ females are fertile [33] and able to suckle their pups. 

 The mammary glands of ERα knockout mice (αERKO), like those of *Sirt1* ^Y/Y^ mice, show reduced ductal outgrowth [38]. Estrogen is a key regulator of mammary gland development [39] and has been shown to promote tumor growth in MMTV-PyMT mice [40]. The connection between the SIRT1 and ERα is confusing. Inhibition of SIRT1 deacetylase activity has been reported to suppress ERα transcription [41] whereas another report showed that SIRT1 repressed estrogen signaling and ERα-mediated cell growth in breast cancer cells *in vitro* [42]. Elangovan et al reported that ERα increases *Sirt1* transcription and that this is essential for estrogen to promote mammary tumorigenesis [43]. In our mice, regardless of *Sirt1* genotype, mammary tumors all had similar levels of SIRT1 and ERα protein levels as assessed by immunohistochemistry, suggesting that absence of SIRT1 catalytic activity does not appear to affect ERα expression in this context. 

 There are several reports indicating that SIRT1 is involved in processes thought to be important for tumor progression and metastases. For example, SIRT1 is reported to modulate growth and invasion [44], neoangiogenesis [45], cell motility [46], epithelial-to-mesenchymal transition [47], and expression of matrix metalloproteinases (MMPs) [48,49]. Nevertheless, tumors arising in the mammary glands of mice containing no SIRT1 catalytic activity are capable of growing aggressively and metastasizing to the lung suggesting that the modulation of these functions is only conditionally dependent on SIRT1. In breast cancer, extremely limited clinical evidence suggests that SIRT1 expression is associated with poorer prognosis [28,29], implying that SIRT1 has a pro-oncogenic effect. This inference is consistent with our result reported above as well as the developing general notion that the biological role of SIRT1 is manifest only under circumstances requiring cellular adaptation to a stress [18]. Breast cancer is, however, a heterogeneous disease with numerous molecular subtypes, and the MMTV-PyMT mouse model is not representative of all of these. Further studies investigating whether the results obtained here were also observed in breast cancer models with different molecular and pathological signatures is warranted.

 Although the results reported in this communication are consistent with our previous work [16] that found that SIRT1-null mice were not differentially sensitive to oncogenic treatments, these results are at odds with other reports [8,9] that suggest that SIRT1 has tumor suppressive properties. For example, the report from Wang et al [9] found that mice heterozygous for a *Sirt1* deletion have enhanced cancer susceptibility and that SIRT1 plays a role in genome stability. One explanation for the conflicting observations might emerge from consideration of the scale-free network of proteins in which SIRT1 is a hub [18]. One might imagine that the line of mice carrying the *Sirt1* ^*-*^ allele created by Wang et al [9] carries another unknown mutation that is synthetic with SIRT1-deficiency to predispose to cancer. Alternatively, the mice carrying the *Sirt1* ^*Y*^ allele described here might carry a different unknown mutation that synthetically suppresses a possible pro-oncogenic property of SIRT1-deficiency. In either case, it seems clear that the effect of compromising SIRT1 function is importantly dependent on genetic context. It may be important to note that both *Sirt1* mutations (and many other genetic modifications to mouse strains) were created by gene knock-in strategies in embryonic stem (ES) cells growing in culture where there is likely to be strong selective pressures for rapid growth and survival. ES cells that acquire mutations in genes that enhance growth or survival would introduce these into the murine germ line and contribute to genetic heterogeneity within the population of animals being compared. 
